# Evaluation of antigen-induced synovitis in a porcine model: Immunological, arthroscopic and kinetic studies

**DOI:** 10.1186/s12917-017-1025-4

**Published:** 2017-04-07

**Authors:** Francisco-Javier Vela, Francisco-Miguel Sánchez-Margallo, Rebeca Blázquez, Verónica Álvarez, Raquel Tarazona, M. Teresa Mangas-Ballester, Alejandro Cristo, Javier G. Casado

**Affiliations:** 1 Stem Cell Therapy Unit, Minimally Invasive Surgery Centre, 10071, Caceres, Spain; 2 Immunology Unit, Department of Physiology, University of Extremadura, 10071, Caceres, Spain; 3 Anaesthetic Unit, inimally Invasive Surgery Centre, 10071, Caceres, Spain; 4 Interactive 3D Unit, Minimally Invasive Surgery Centre, 10071, Caceres, Spain; 5 CIBER de Enfermedades Cardiovasculares, Caceres, Spain

**Keywords:** Synovitis, Animal model, Inflammation

## Abstract

**Background:**

Synovitis is an inflammation-related disease linked to rheumatoid arthritis, osteoarthritis, infections and trauma. This inflammation is accompanied by immune cells infiltration which initiates an inflammatory response causing pain, discomfort and affecting the normal joint function. The treatment of synovitis is based on the administration of anti-inflammatory drugs or biological agents such as platelet rich plasma and mesenchymal stem cells. However, the evaluation and validation of more effective therapies of synovitis requires the establishment of clinically relevant animal models.

**Results:**

In this study, Large White pigs were pre-immunized to evaluate an antigen-induced synovitis. The immune monitoring of synovial fluids in this model allowed us the identification of IL-12p40 and T cell subsets as immune biomarkers. Moreover, the evolution of synovitis was performed by arthroscopic procedures and kinetic analysis. In summary, this paper describes an animal model of antigen-induced synovitis to be used in the evaluation of anti-inflammatory therapies.

**Conclusions:**

The novelty of this paper lies in the development of a clinically relevant model of synovitis which permits the simultaneous evaluation of synovitis from an immunological, surgical and kinetic point of view.

**Electronic supplementary material:**

The online version of this article (doi:10.1186/s12917-017-1025-4) contains supplementary material, which is available to authorized users.

## Background

The synovial fluid (SF) is a transudate of plasma that provides low-friction for a normal joint function [1]. The homeostasis of SF depends on the continuous renewal from the lymphatic capillaries to the articular cavity and the transynovial filtration towards the lymphatic capillaries. This renewal is also facilitated by physical exercise and joint flexion [2]. The synovitis is an inflammation-related disease usually linked to rheumatoid arthritis [3], osteoarthritis [4, 5] and viral infections [6]. The inflammation of synovial tissue is accompanied to immune cells infiltration (mainly composed by macrophages and T and B lymphocytes) which initiates an inflammatory response causing pain and discomfort [7] and affecting the normal joint function [8].

At the present, some of the most common treatments for synovitis are based on the administration of non-steroidal anti-inflammatory drugs [9] as well as biological agents such as platelet rich plasma [10], autologous conditioned medium [11] and mesenchymal stem cells isolated from different sources [12] that have become a promising therapeutic option in regenerative medicine due to their self-renewal capacity, multipotentiality and immunomodulatory properties [13, 14].

Similarly to other diseases, the development of more effective therapies for the treatment of synovitis requires the establishment of clinically relevant animal models. Ideally, a valuable animal model for this inflammatory-related disease should be suitable for the evaluation of immune biomarkers and kinematic parameters. At the present, several animal models of synovitis have been described including dogs [15], rats [16] and rabbits [17]. However, due to their translational applicability, other large animal models such as pigs or sheep have been considered as the more appropriate models for this kind of studies [18]. Concretely, the morphological and physiological similarities of pigs and humans in terms of cartilage thickness, biomechanical features and joint dimensions become this animal model particularly attractive for further clinical translation.

Based on that, the aim of this work was to develop and characterize a clinically relevant large animal model of synovitis. The novelty of this manuscript lies in the development of an animal model which permits to researchers the evaluation, monitoring and follow up of synovitis from different perspectives: firstly, from an immunological point of view through the evaluation of immunological biomarkers (synovial fluid lymphocytes and IL-12p40), and secondly, from a biomechanical and surgical point of view by arthroscopic and kinetic gait analysis.

## Methods

### Animals and experimental design

Eight Large White pigs were housed in the animal facility at the Minimally Invasive Surgery Centre and used for all experimental procedures. Animals aged 3 months and weighed 25–35 kg at the beginning of the study were used. All experimental protocols were approved by the Committee on the Ethics of Animal Experiments of Minimally Invasive Surgery Centre and fully complied with recommendations outlined by the local government (Junta de Extremadura) and by the Directive 2010/63/EU of the European Parliament on the protection of animals used for scientific purposes.

All the animals were pre-immunized by subcutaneous injections of bovine serum albumin (BSA). Local immunizations for synovitis induction were performed by intra-articular injection of BSA on the right carpal joint of each animal. The left carpal joints received an intra-articular injection of phosphate buffer saline (PBS) to be used as negative control.

As an additional control group, three Large White pigs without BSA pre-immunization were included in this study. Intra-articular injections of PBS and BSA were performed in the left and the right carpal joints respectively.

### Anesthetics procedures

Every procedure was done under anesthesia. For blood sampling and subcutaneous BSA injections, anesthesia was induced by intramuscular injection of 10 mg/kg ketamine hydrochloride and 0.02 mg/kg dexmedetomidine hydrochloride. The animals were recovered with 0.02 mg/kg atipamezole hydrochloride. For SF sampling and arthroscopies, anesthesia was induced by the same procedure together with 2 mg/kg propofol on intravenous bolus injection, and the analgesia was performed with 3 mg/kg of tramadol. According to ethical and animal welfare concerns, all the animals received analgesic treatment with buprenorphine hydrochloride. The buprenorphine at 0.3 mg/ml was regularly administered at 0.03 ml/kg for 7 days after intra-articular injection.

### Immunization protocol

**Fig. 1:**
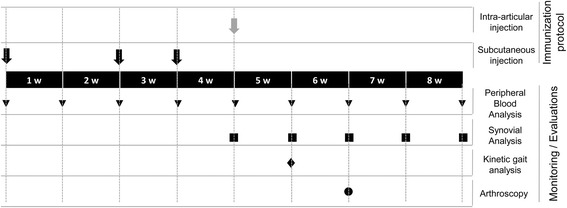
Temporal scheme of the immunization protocol and monitoring. Subcutaneous BSA injections (*black arrows*), intra-articular BSA injection (*grey arrow*), blood sampling (*triangles*), synovial fluid sampling (*squares*), pressure platform gait analysis (*rhombus*) and the arthroscopic surgery (*circle*) are shown

For animal immunizations, a solution with 20 mg/ml of BSA (Sigma-Aldrich, St. Louis, MO, USA) was prepared and passed through a 0.2 μm sterilized microfilter. An equal volume of Freund Complete Adjuvant (FCA) (Sigma-Aldrich, St. Louis, MO, USA) was mixed with the BSA solution and emulsified. The immunization was performed by subcutaneous injection of this emulsion. A total of 0.4 ml/kg was injected on days 0, 14 and 21 (see Fig. 1). On day 28, a total of 0.5 ml of SF was aspirated from both joints (SF basal sample) and intra-articular immunizations of BSA were performed in the forelimbs. A total of 0.5 ml of BSA at 20 mg/ml was injected on the right carpal joint to induce the synovitis and 0.5 ml PBS on the left carpal joint (used as negative control).

### Isolation and phenotypic characterization of synovial fluid and peripheral blood lymphocytes

Synovial fluid leukocytes (SFLs) were obtained from carpal joints. A total of 0.5–1 ml of SF was aspirated and weekly sampled for 5 weeks (see Fig. 1). Leukocytes were counted in an automatic hematology analyzer (Mindray BC-5300 Vet, Hamburg, Germany) and SFLs were isolated by centrifugation at 900×g and used for flow cytometric analysis. Supernatants were also collected and stored at −20 °C for cytokines determination.

Peripheral blood lymphocytes (PBLs) were obtained from jugular vein blood samples. Blood sampling was performed weekly from the beginning of the study. PBLs were isolated by centrifugation over Histopaque-1077 (Sigma-Aldrich) and washed twice with PBS for cytometric analysis.

For flow cytometric analysis, PBLs and SFLs were suspended in PBS containing 2% FBS. The cells were then stained with PerCP-conjugated monoclonal antibody against porcine CD4 (Mouse Anti-Pig CD4a, clone: 74–12-4, BD Pharmingen, San Jose, CA, USA) and APC-conjugated monoclonal antibody against porcine CD8 (Mouse Anti-Pig CD8a, clone: 76–2-11, BD Pharmingen). The cytometric analysis was performed as follows: 2 × 10^5^ cells were incubated for 30 min at 4 °C with appropriate concentrations of monoclonal antibodies. The cells were washed and resuspended in PBS. The flow cytometric analysis was performed in a FACScalibur cytometer (BD Biosciences) after acquisition of 10^5^ events. Cells were primarily selected using forward and side scatter characteristics and fluorescence was analyzed using CellQuest software (BD Biosciences, San Jose, CA, USA). Isotype-matched negative control antibodies were used in all the experiments.

### Quantification of anti-BSA antibodies by ELISA

In order to quantify the anti-BSA IgG titers on immunized animals, an ELISA test was performed on plasma samples. Microplate coating was performed by an overnight incubation with BSA at 20 μg/ml. The next day, coating solution was removed and wells were washed twice with 200 μl of PBS/Tween-20 (0.05%, 7.4 pH). In order to prevent the nonspecific binding of the antibodies, the remaining protein-binding sites were blocked by adding 200 μl of BSA and the plate was incubated for 2 h at 4 °C. The plate was washed four times with 200 μl PBS/Tween-20. Plasma samples were diluted on PBS at 1/200 and 100 μl of this dilution was added to each well. The plate was incubated for 2 h at 4 °C. After washing four times with PBS/Tween-20, 100 μl of 1/5000 diluted horseradish peroxidase (HRP) -conjugated secondary antibody (Rabbit Anti-Pig IgG, Thermo Fisher Scientific, Waltham, MA, USA) were added to each well and the plate was incubated for 2 h at 4 °C. Again, the plate was washed four times and 100 μl of the enzyme substrate (3,3′, 5,5′-Tetramethylbenzidineor TMB, Sigma-Aldrich) were added to each well. Two minutes later, 100 μl of 1 N HCl were added *per* well in order to stop the reaction. Plate absorbance was measured at 450 nm on a Synergy Mx spectrophotometer (BioTech Industries, Newton, NC, USA).

### Cytokine detection and measurement with multiplex technology

The supernatants of SF were diluted 1:4 in PBS and stored at −20 °C. These supernatants were thawed and IFNα, IFNγ, IL-1b, IL-4, IL-6, IL-8, IL-10, IL-12p40 and TNFα were analyzed using a multiplexed immunoassay. The measurements were performed according to the manufacturer’s instructions by Luminex xMAP technology using the ProcartaPlex Porcine Cytokine & Chemokine Panel 1 (eBioscience, San Diego, CA, USA; catalog number EPX090–60829-901). The concentrations of the different cytokines were expressed as pg/ml, and calculated according to a standard curve.

### Arthroscopies

In order to evaluate the potential changes on the joint status, arthroscopies were performed in the carpal joints of pre-immunized animals at two weeks after intra-articular PBS or BSA injection. The arthroscope used on surgical procedures was a HOPKINS® wide angle forward-oblique telescope 30°, 2.4 mm diameter, 10 cm length (Karl Storz, Tuttlingen, Germany).

A needle was used as a guide for the correct placement of the arthroscope through a small incision. A saline flux through the joint was maintained during all the procedure to provide a better visualization of the tissues. A careful and detailed evaluation of the joint was performed and photo recorded. Finally, the 3 mm incision was closed with a 2–0 absorbable suture and every animal received antibiotic treatment (clavulanic acid + amoxicillin) during 7 days after arthroscopy.

### Pressure platform gait analysis functional evaluation by biomechanical analysis

A 174.5 cm × 36.9 cm pressure platform (PP) (Walkway™; Tekscan, South Boston, MA, USA), composed by individual sensors with a density of 1.4 sensor/cm^2^ and 9152 sensors in total, was used for the biomechanical evaluation. The sensors of the PP walkway were calibrated according to the manufacturer’s specifications. Seven days after intra-articular injection, animals were guided to walk along the PP and after at least 5 complete passes *per* animal, data were analyzed. Impulse (kg x sec) and vertical maximum force (kg) were determined. Measurements were normalized to animal weights.

### Statistical analysis

Data were statistically analyzed using the non-parametric Man Whitney *U*-test for paired comparisons and Kruskal-Wallis test for multiple comparisons. The *p*-values ≤0.05 were considered statistically significant. All the statistical determinations were made using SPSS-21 software (SPSS, Chicago, IL, USA).

## Results

### The BSA-immunization protocol elicits an antibody and T cell response on porcine model

The animals were subcutaneously immunized with an emulsion of BSA and FCA on days 0, 14 and 21. During the immunization protocol, peripheral blood was weekly collected from vein and analyzed by flow cytometry to evaluate the percentage of CD4 + T cells, CD8+ T cells and their ratio. It is important to note that anti-CD4 and anti-CD8 antibodies were simultaneously used for the quantification of CD4+/CD8- and CD4−/CD8+ subsets. The presence of anti-BSA antibodies in plasma samples was also quantified by ELISA test.

**Fig. 2:**
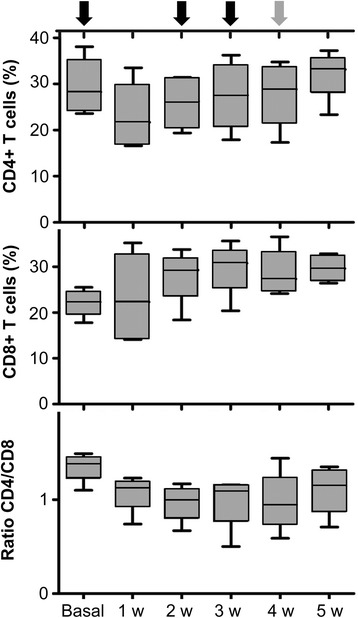
Lymphocyte subsets distribution in peripheral blood cells from BSA-immunized animals. Peripheral blood lymphocytes were weekly collected for flow cytometry analysis. Black arrows indicate the subcutaneous BSA injections and the grey arrow indicates the intra-articular injection. The graphic shows the percentage of CD4+ T cells, CD8+ T cells and their ratio. The lower boundary of the box indicates the 25th percentile and the upper boundary the 75^th^percentile. Bars above and below the box indicate the 90th and 10^th^ percentiles. The line within the box marks the median (*n* = 4). No statistically significant differences were found between groups

The analysis of peripheral blood lymphocytes from BSA-immunized animals demonstrated that the CD8 + T cell subset showed a trend to increase (non-statistically significant) whereas both CD4 + T cell subset and CD4/CD8 ratio showed a trend to decrease (Fig. 2).

**Fig. 3:**
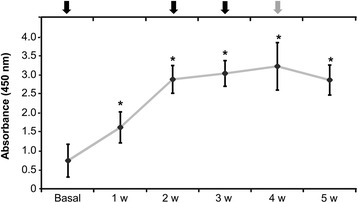
Humoral response to bovine serum albumin in immunized animals. Plasma samples were weekly collected and anti-BSA IgG levels were quantified by ELISA immunoassay. In the graphic, *black* arrows indicate the subcutaneous BSA injections and the *grey* arrow indicates the intra-articular injection. Values show the mean ± SD (*n* = 4). *Statistically significant difference (*p* ≤ 0.05) compared to basal level

Regarding the evaluation of antibodies in plasma samples, our results demonstrated that anti-BSA IgG antibody titers were detected in all of the four animals. The antibody concentrations significantly increased when compared 7 and 14 days and remained stable from days 14 to 35 showing a maximum level at 4 weeks (Fig. 3).

Based on these immunoassays, here we demonstrate that BSA-immunization protocol triggered the pre-sensitization of this animal model, which is prerequisite to generate an antigen-induced synovitis.

### Intra-articular administration of BSA on pre-immunized animals modifies the leukocyte counts and synovial lymphocytes distribution

The pre-sensitized animals (subcutaneously immunized with BSA at day 0, 14 and 21) received an intra-articular injection of PBS or BSA in left or right carpal joints, respectively, at day 28. Basal samples were aspirated at day 28 prior to PBS or BSA injections. Synovial fluids were aspirated at days 35, 42, 49 and 56 (Fig. 1). The synovial fluids were centrifuged and synovial leukocytes were processed for flow cytometry analysis. Non-cellular fraction of synovial fluid was frozen for subsequent cytokine analyses.

The counting of leukocytes from synovial fluid samples demonstrated that, at day 35 (7 days post intra-articular BSA), the leukocyte counts were significantly increased (*p* = 0.04) in those carpal joints where BSA was intra-articularly injected: 0.75 ± 1.12 × 10^6^ /ml in control samples vs 2.40 ± 1.19 × 10^6^/ml in BSA-injected.

Moreover, the lymphoid and myeloid synovial cells were quantified in an automatic hematology analyzer. The distribution of lymphoid/myeloid cells in control samples was: 67.95 ± 6.57 (% of lymphoid cells) and 32.05 ± 8.11 (% of myeloid cells). On the other hand, the distribution of lymphoid/myeloid cells after intra-articular BSA injections was: 40.9 ± 19.93 (% of lymphoid cells) and 59.1 ± 20.79 (% of myeloid cells).

**Fig. 4:**
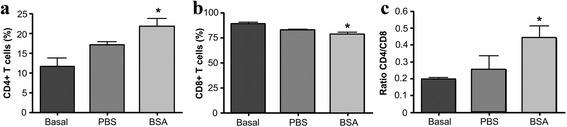
Distribution of synovial lymphocyte subsets. Synovial fluid lymphocytes were collected for flow cytometric analysis just before intra-articular injection (basal) and 7 days after. The graphic shows the percentage of CD4+ T cells (**a**) CD8+ T cells (**b**) and their ratio **c**. Values show the mean ± SD (*n* = 3). *Statistically significant difference (*p* ≤ 0.05) compared to basal level

Once demonstrated that leukocyte counts were significantly increased, the analysis of synovial lymphocytes CD4 + T cells, CD8 + T cells and their ratio was performed at day 7, 14 and 21 after intra-articular BSA or PBS injections. Our results did not show any significant difference at days 14 and 21 (data not shown). In contrast, significant differences were observed when synovial lymphocytes were quantified at day 7 after intra-articular BSA injections (Fig. 4). As shown in Fig. 4, the intra-articular administration of BSA on pre-immunized animals exerted a significant decrease of synovial CD8 + T cells when compared to basal values (*p* = 0.025). In contrast, the percentage of CD4 + T cells as well as the CD4/CD8 ratio was significantly increased (*p* = 0.025 and *p* = 0.026, respectively). It is important to note that, in order to have a control for intra-articular injections, PBS was intra-articularly injected in pre-sensitized animals and no significant differences were observed when compared to basal values (Fig. 4). Moreover, in order to establish a proper negative control, PBS and BSA were intra-articularly injected in non-immunized animals. No differences were observed in terms of CD4 + T cells, CD8 + T cells, CD4/CD8 ratio (Additional file 1) and biochemical parameters (Additional file 2).

Altogether, our results demonstrated that intra-articular administration of BSA on pre-immunized animals elicited a significant increase of synovial leukocytes as well as a redistribution of synovial T cell subsets towards a CD4-driven response.

### Local administration of BSA on pre-sensitized animals modifies the cytokine profile of synovial fluid

Once evaluated the changes in the leukocyte counts as well as in the percentages of synovial CD4+ and CD8+ T cells, we aimed to evaluate the inflammatory environment by quantifying a wide range of cytokines. The following cytokines were quantified by Luminex technology: IFNα, IFNγ, IL-1b, IL-4, IL-6, IL-8, IL-10, IL-12p40 and TNFα.

The synovial fluids from immunized animals only showed detectable and significant differences on IL-12p40 cytokine (data not shown for the rest of cytokines). This cytokine was quantified at days 7, 14 and 21 after intra-articular BSA injections.

**Fig. 5:**
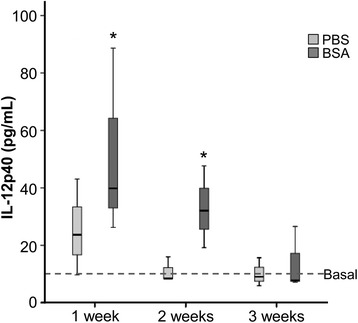
Quantification of IL-12p40 levels in synovial fluid. Synovial fluid was collected at day 7, 14 and 21 after intra-articular injection of PBS and BSA. Cytokine levels were determined by Luminex xMAP technology. The lower boundary of the box indicates the 25th percentile and the upper boundary the 75th percentile. Bars above and below the box indicate the 90^th^ and 10^th^ percentiles. The line within the box marks the median (*n* = 4). Dot line indicates the basal levels (just before intra-articular injection). *Statistically significant difference (*p* ≤ 0.05) compared to basal level

Our results showed significant differences on IL-12p40 at day 7 after intra-articular BSA-immunization (*p* ≤ 0.05) and non-significant differences (but a trend to increase) were found at day 14. Finally, non-significant differences were observed after 21 days (Fig. 5).

### Arthroscopy as a diagnostic procedure in synovitis

**Fig. 6:**
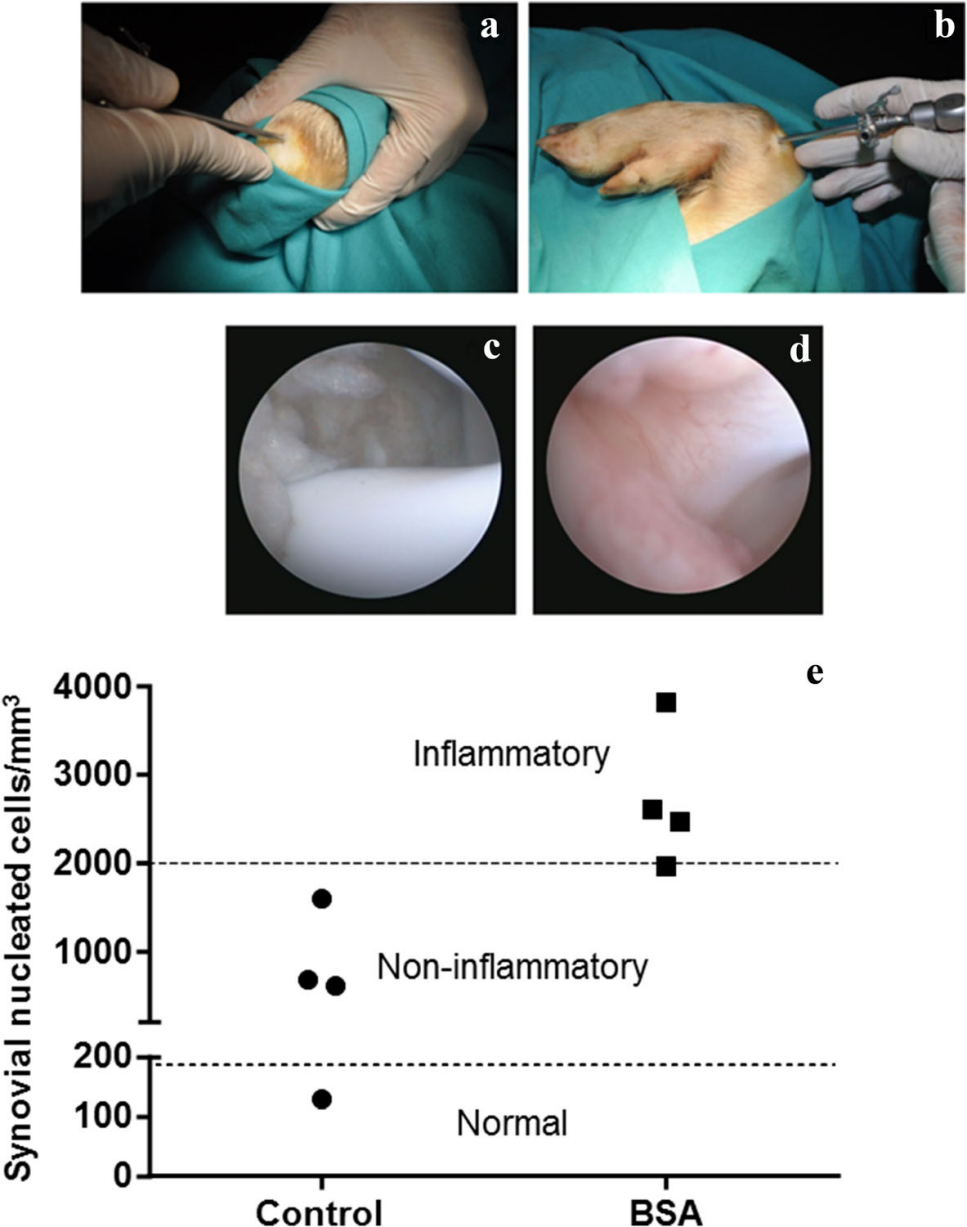
Surgical approach and arthroscopic analysis. Two weeks after intra-articular injection of PBS or BSA, an arthroscopic evaluation was performed. Figure shows the access to the articular cavity **a**, the arthroscopic procedure **b** representative image of arthroscopy in the control joint **c** and representative image of arthroscopy in the BSA-injected joint **d**. Synovial fluid classification according to nucleated cells/mm^3^ **e**. Synovial fluid is classified as “normal” if it contains less than 180 nucleated cells/mm^3^ or “non-inflammatory” when synovial fluid contains less than 2000 cells/mm^3^. On the contrary, when synovial fluid contains 2000–50,000 cells/mm^3^ it is classified as “inflammatory” [19]

Carpal joints from BSA-immunized animals were evaluated by minimally invasive procedures. Arthroscopy was performed at day 14 after intra-articular BSA or PBS injections. A total of 8 arthroscopic evaluations were performed and four out of four carpal joints where BSA was intra-articularly injected showed a slightly red to orange color (Fig. 6d). In contrast, those control carpal joints where PBS was injected, showed clear, colorless or straw colored synovia (Fig. 6c).

Apart from the macroscopic observation, aspirated synovial fluids from control and BSA were classified according to synovial nucleated cells. This classification is based on a previous report from El-Gabalawy [19] where synovial fluid can be classified as Normal, if it contains fewer than 180 nucleated cells/mm^3^; Non-inflammatory, when synovial fluid contains less than 2000 cells/mm^3^, and Inflammatory, when synovial fluid contains 2000–50,000 cells/mm^3^. Our results demonstrated that, synovial fluid from control joints can be classified as Normal or Non-inflammatory and those synovial fluids where BSA was injected could be considered as Inflammatory (Fig. 6e).

### Monitoring of synovitis by pressure platform gait analysis

**Fig. 7:**
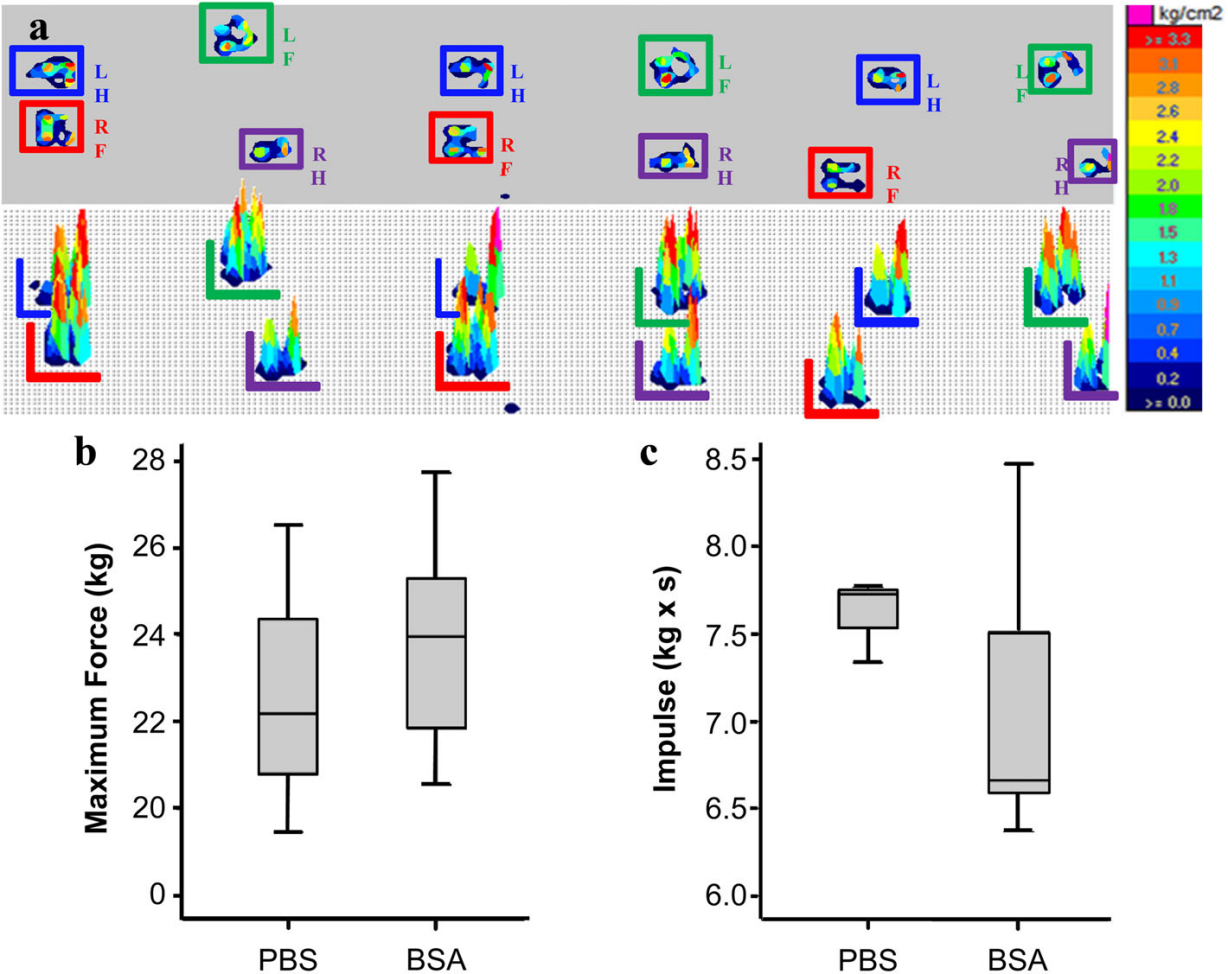
Pressure platform gait analysis. Seven days after intra-articular injection of PBS or BSA, a pressure platform gait analysis was performed to evaluate plantar pressure distributions. **a** Above, a representative image of the gait analysis (*LF*: *left forelimb*; *LH*: *left hind limb*; *RF*: *right forelimb*; *RH*: *right hind limb*) is represented. Below, the pressure of each limb is shown. The legend on the right shows the equivalence between numeric and colorimetric values. Maximum forces **b** and impulses **c** in control and BSA-injected limbs (*n* = 4). The lower boundary of the box indicates the 25^th^ percentile and the upper boundary the 75th percentile. Bars above and below the box indicate the 90^th^ and 10^th^ percentiles. The line within the box marks the median. No significant differences were found between PBS and BSA injected joints

The kinematic gait parameters were evaluated by a pressure platform. The pre-sensitized animals were biomechanically evaluated at day 7 after intra-articular injections of BSA or PBS. The parameters evaluated were impulse (Kg x sec) and the vertical maximum force (Kg). In terms of animal management, our experience demonstrated that kinematic parameters could be easily quantified with the porcine model (Fig. 7a). Our results showed that, the impulses in the forelimbs with BSA showed an enormous inter-individual variability (Fig. 1c) and no significant difference was observed in terms of vertical maximum force (Fig. 7b). Finally, it is important to note that because of ethics and animal welfare, this kinematic analysis had to be performed under analgesia.

## Discussion

Synovitis is an inflammation of the joint lining. This inflammation is painful and usually linked to osteoarthritis, rheumatoid arthritis or infections [3, 4, 6]. To alleviate pain and discomfort, the synovitis can be successfully treated with anti-inflammatory medications such as non-steroidal anti-inflammatory drugs [9] or biological therapies [10, 11, 14, 20]. At the present, new therapies are currently being investigated to improve their clinical efficacy and to reduce the adverse effects commonly associated to non-steroidal anti-inflammatory drugs.

Clinically relevant animal models are essential to evaluate therapeutic strategies to target inflammation and to predict outcome of clinical trials. The anatomical similarities between pigs and humans, particularly for surgical procedures, makes this animal model a valuable tool to evaluate the safety, feasibility and dosage pattern of new therapies for synovitis. Based on that, the main objective of our work has been focused in the development of an experimentally-induced synovitis model to be used for the evaluation and follow up of synovitis from different perspectives. It is important to note that, the synovitis in the animal is triggered by a T cell-mediated response induced by BSA, which is somehow comparable to the T cell-mediated response in the synovial tissue of clinically active rheumatoid arthritis patients [21].

It is interesting to note that immunologically induced synovitis models have been previously described in pigs. In this sense, Möller et al. succeeded in inducing a reliable and reproducible synovitis using turkey egg albumin (more appropriate than chicken egg albumin) [18]. This animal model was proposed to be useful to investigate the potential applicability of laser treatment in arthroscopic synovectomy. In contrast to our BSA-induced synovitis model, in the turkey egg albumin model from Möller et al. the synovitis examination was performed in terms of macroscopic observations (joint profile, synovial fluid and membrane) as well as in terms of histologic findings (synovial membrane, stratum synoviale and fibrosum). Our antigen-induced synovitis model provides the methodologies and procedures for monitoring the inflammation in terms of immune biomarkers. Moreover, similarly to the model from Möller et al. this animal model allowed us the evaluation of synovitis by arthroscopic inspection.

First of all, the BSA pre-sensitization in the porcine model was optimized and adapted taking as a reference previous reports with rabbits and dogs [22–24]. Similarly to antigen-induced synovitis model developed in other animals, our results demonstrated that BSA emulsified with complete Freund’s adjuvant was strongly efficient to promote a rapid and maintained antigen-specific humoral response. Moreover, based on the kinetic of the humoral response against BSA, and taking into account that the anti-BSA titers did not significantly increased after second and third immunizations, it would be interesting to evaluate in future experiments a different pre-sensitization protocol based on a single BSA-immunization. Apart from humoral response against BSA, and in contrast to other studies using antigen-induced synovitis models, here we focused our interest on the cellular response. In this sense, we analyzed the CD4+ and CD8+ T cell subsets on the peripheral blood from BSA-immunized animals. Our results demonstrated that BSA-immunization induced slight changes (non-statistically significant) on T cell subsets. However, the absence of significant changes would be the consequence of analgesic and anti-inflammatory treatment with buprenorphine (required by ethical guidelines) during immunization.

Once demonstrated that the immunization protocol triggered a humoral response against BSA, we aimed to induce an inflammatory response in synovial tissue. Our results demonstrated that, intra-articular administration of BSA significantly induced changes on synovial leukocyte counts. Moreover, the flow cytometry analysis showed significant changes in the distribution of synovial CD4+ and CD8+ T cells. Based on that, and taking into account that the presence of large numbers of activated CD4+ T cells in synovial tissue is important in the pathogenesis of chronic synovitis [25], here we suggest that synovial T cell distribution could be suitable biomarker in the evolution of synovitis.

Additionally, although the functional phenotype of synovial CD4+ T cells could not be evaluated (because of the limited availability of synovial lymphocytes), the significant increase of synovial CD4+ T cells may reflect an increase of ‘type 1’ polarity (TH1) with CD4+ CD45RO+ phenotype and IFN**γ** expression. This assumption is based on previous reports which demonstrated that synovial CD4+ T cells from rheumatoid arthritis patients were predominantly of ‘type 1’ polarity [26–28].

Additionally, in order to identify soluble factors to be used as synovitis biomarker in this animal model, we aimed to characterize a large panel of cytokines in the synovial fluids from immunized animals. It is important to note that, from an immunological and mechanistic point of view, it was important to define if local inflammatory response is dominated by a Th1 or Th2 response [29]. Based on that premise, the following panel of cytokines was quantified: IFNα, IFNγ, IL-1b, IL-4, IL-6, IL-8, IL-10, IL-12p40 and TNFα. Our results showed that, probably because of the detection limit of commercially available swine immune reagents, only IL-12p40 could be efficiently quantified in the synovial fluids. The IL-12p40 is a subunit of IL12p70 [30] which is mainly produced by monocytes, macrophages and dendritic cells [31]. The presence of this cytokine initiates the differentiation of Naïve CD4+ T cells towards a Th1 response [32], so according to the observed increase of IL-12p40, here we hypothesize that this antigen-induced synovitis model is dominated by a local Th1 response.

It is important to discuss the importance of synovial biomarkers in the evaluation of the disease course and monitoring of treatments. In this sense, several inflammatory cytokines have been defined as biomarkers in patients with rheumatoid arthritis and osteoarthritis [33]. Other authors have also identified the Cartilage Oligometric Matric Proteinin in synovial fluid as a biomarker to predict the development of osteoarthritis [34]. The levels of C-terminal telopeptides of type II collagen and hyaluronan have also been defined as potential biomarkers of synovitis [35, 36]. Here we hypothesize that IL-12p40 could be considered as immunological biomarker in the evaluation of synovitis in this animal model. Supporting this statement, similar changes have been observed in the synovial tissue of rheumatoid arthritis patients where increased IL-12p40 levels have been detected [37].

Apart from the identification of immunological biomarkers in the evaluation of disease progression, this paper aimed to have a multi-criteria assessment in the evaluation of synovitis. Arthroscopic evaluations and pressure platform gait analyses were performed to have additional elements in the monitoring of synovitis. Firstly, arthroscopy was found to be a simple and feasible surgical technique to evaluate the synovitis in this animal model. Additionally, the kinematic parameters quantified in the animal model were a valuable tool to evaluate the functional parameters (impulse and vertical forces). This porcine model, in contrast to the sheep that become nervous and more difficult to handle [38], demonstrated several advantages in terms of animal handling being particularly suitable for gait analysis. Unfortunately, our results did not reveal any significant difference in terms of impulse or vertical forces when forelimbs were compared in the animal model. In this case, the absence of differences would not necessarily imply that the kinetic was unaffected, so we should clarify that ethical consideration and animal welfare guidelines forced us to perform the pressure gait analysis in animals under analgesia.

## Conclusions

Taking into consideration that the establishment of an animal model is an absolute prerequisite in preclinical research, this paper describes the development and characterization of a clinically relevant porcine model of synovitis. This antigen-induced inflammation model triggered a cell-mediated response allowing us the identification of immunological parameters to be used as biomarkers in the monitoring of synovitis and newly developed therapies. Moreover, here we demonstrated that, our antigen-induced model of synovitis can also be evaluated by standard arthroscopic instruments and kinetic studies.

## Additional files

Additional file 1: Distribution of synovial lymphocyte subsets in control samples. Synovial fluid lymphocytes were collected from non pre-immunized animals. Flow cytometric analysis was performed on synovial fluids at day 7 after PBS (0.5 ml) or BSA injections (0.5 ml of BSA at 20 mg/ml). The graphic shows the percentage of CD4+ T cells (A), CD8+ T cells (B) and their ratio (C). Values show the mean ± SD (*n* = 3). (JPEG 154 kb)

Additional file 2: Biochemical analysis of synovial fluid in non-pre-immunized animals (*n* = 3). (DOCX 14 kb)

## Abbreviations

BSA: Bovine Serum Albumin
FCA: Freund Complete Adjuvant
MSCs: Mesenchymal Stem Cells
PBLs: Peripheral Blood Lymphocytes
PBS: Phosphate Buffer Saline
SF: Synovial Fluid
SFLs: Synovial Fluid Lymphocytes
Th1: T helper 1
