# Supplementary figures and images for: Evaluation of antigen-induced synovitis in a porcine model: Immunological, arthroscopic and kinetic studies
Source: BMC Vet Res. 2017 Apr 7;13:93. doi: 10.1186/s12917-017-1025-4 (PMC5384159; doi:10.1186/s12917-017-1025-4)

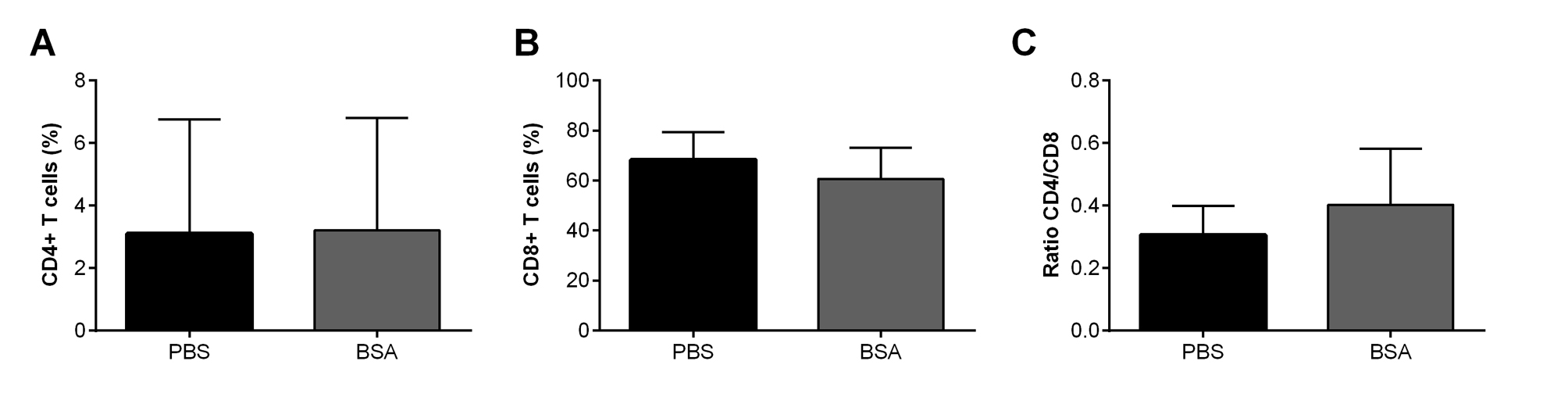

Supplement: Supplementary file 1 — Distribution of synovial lymphocyte subsets in control samples. Synovial fluid lymphocytes were collected from non pre-immunized animals. Flow cytometric analysis was performed on synovial fluids at day 7 after PBS (0.5 ml) or BSA injections (0.5 ml of BSA at 20 mg/ml). The graphic shows the percentage of CD4+ T cells (A), CD8+ T cells (B) and their ratio (C). Values show the mean ± SD (n = 3). (JPEG 154 kb) [file 12917_2017_1025_MOESM1_ESM.jpg]
