# Supplementary material for: Evaluation of antigen-induced synovitis in a porcine model: Immunological, arthroscopic and kinetic studies
Source: BMC Vet Res. 2017 Apr 7;13:93. doi: 10.1186/s12917-017-1025-4 (PMC5384159; doi:10.1186/s12917-017-1025-4)
Supplement: Supplementary file 2 — Biochemical analysis of synovial fluid in non-pre-immunized animals (n = 3). (DOCX 14 kb) [file 12917_2017_1025_MOESM2_ESM.docx]

|  | **PBS**  **(Mean ± SD)** | | **BSA**  **(Mean ± SD)** | |
| --- | --- | --- | --- | --- |
| **Glucose (mg/dl)** | 34.67 | ± 23.03 | 43.33 | ± 21.22 |
| **Total protein (g/dl)** | 0.62 | ± 0.19 | 0.80 | ± 0.17 |
| **Urea (mg/dl)** | 28.53 | ± 13.12 | 34.93 | ± 8.50 |

**Additional file 2. Biochemical analysis of synovial fluid in non-pre-immunized animals (n=3).**
